# To realize a variety of structural color adjustments via lossy-dielectric-based Fabry–Perot cavity structure

**DOI:** 10.1515/nanoph-2022-0522

**Published:** 2022-11-03

**Authors:** Md Abdur Rahman, Dong Kyu Kim, Jong-Kwon Lee, Ji Young Byun

**Affiliations:** Extreme Materials Research Center, Korea Institute of Science & Technology, 5, Hwarang-Ro 14-Gil, Seongbuk-Gu, Seoul 02792, Republic of Korea; Division of Energy and Optical Technology Convergence, Cheongju University, Cheongju-Si, Chungcheongbuk-Do 28503, Republic of Korea

**Keywords:** Fabry–Perot cavity, lossy dielectric, reflection colors, strong light absorption, structural colors

## Abstract

Structural colors with tunable properties have extensive applications in surface decoration, arts, absorbers, and optical filters. Planar structures have more advantages over other forms studied to date due to their easy manufacturability. Metal-insulator-metal-based structures are one of the known methods to fabricate structural colors where colors can be tuned mainly by the thickness of the intermediate lossless insulator layer. However, generating colors by MIM structure requires a thin metallic layer on top, and the top metals’ abrasiveness and/or oxidation may degrade the colors quickly. Thus, we propose a lossy dielectric layer to replace the top metallic layer as a solution to ensure the structure’s durability by preventing scratches and oxidation. Herein, CrON/Si_3_N_4_/Metal structures have been studied where theoretical investigations suggest that highly saturated colors can be generated in the lossy-lossless dielectric structures. Experimental data validated such simulations by revealing a range of vivid colors. Furthermore, these structures can easily achieve strong light absorption (SLA) even for a thick top layer of ∼100 nm. The colors realized by these structures are appeared due to a combination of the interference effect of the asymmetric Fabry–Perot cavity structure and the absorption rate in the CrO_*x*_N_1−*x*_ layer.

## 1 Introduction

In the recent past, metal-based structural coloration has exhibited its huge prospect in various applications due to its non-toxicity, non-fading property as well, as esthetic appeal, compared to pigment- or dye-based coloration [1]. To realize structural colors, several designs have been extensively studied, such as bio-inspired structures [2–4], photonic crystals [5–7], amorphous photonic structures [8], diffraction gratings [9], and plasmonic nanostructures [10–13]. Besides these studies, planar structures such as two-layer dielectrics structures [14] and tri-layer metal–insulator–metal (MIM) stack-based Fabry–Perot (F–P) cavities [15–23] have also been reported. The planar structure has more advantages in fabricating structural colors as it does not require sophisticated nano-patterning techniques.

MIM structures provide more freedom than dielectric-based structures among the planar structures, as the former generates more vivid structural colors. While designing a MIM structure, lossy metals, e.g., Au [15–17], Ag [18], Ni [19–21], Al [22], Cu [23], and Cr [24] as the top layer and dielectrics as the intermediate layer are generally chosen. Reflective colors are generated on optically thick metals. A grayscale lithography method is also proposed for dynamic coloration by MgH_2_ acting as top layer. Coloration was performed using stepwise nanocavity creation of hydrogen silsesquioxane (HSQ) layer on an Al reflector [25]. Previous studies also report the typical lossy dielectric e.g., Ge and Si-based absorber [26–28]. The Ge/SiO_2_/Ge structure is proposed as a lithography-free absorber, and these structures are limited to fabricate black/dark blue color coatings [26]. Another lossy dielectric Si based cavity structure is proposed where a range of colors can be fabricated on the AlCu substrate by tuning the thickness of the top layer, Si [27, 28].

In the F–P cavity structures, the thickness of the intermediate layer, *h*_d_ plays a pivotal role in tuning the structural color as the phase condition is directly related of *h*_d_. Though the MIM-based structural colors provide a range of colors, a few issues of this design remain. Firstly, the top layer is chosen as metal which is easily abrasive and/or oxidized in the long run. Secondly, to design the highly vivid colors, the thickness of the top layer should be limited to <30 nm depending on the metal’s lossy property.

Previous studies suggest that CrN and CrN-based surfaces provide good corrosion resistive properties over the whole structures [29–32]. The optical property of CrN behaves as lossy dielectric. In this study, we have conducted a comparative study of CrN and CrON-top layer based Fabry Perot structure as an alternative to typical MIM structure as an F–P structure. The trend of reflectance and colors for the CrN-based structure is similar to a typical MIM structure, but reflectance and colors obtained from the CrON-based structure are way more promising. Besides the theoretical predictions of structural colors, experimental works were also carried out as evidence.

## 2 Materials and methods

To fabricate the CrN or CrO_*x*_N_1−*x*_/Si_3_N_4_/Al (and Cu) tri-layer, an optically thick Al and Cu layer was first deposited onto a polished (111) Si wafer using an electron-beam evaporator (EBX-1000, ULVAC, Japan). After evacuating the chamber to a base pressure of 3 × 10^−7^ Torr, the deposition was carried out. An optically thick STS layer was used as a back reflector for fabricating CrN or CrO_*x*_N_1−*x*_/Si_3_N_4_/STS structure, and STS thin film was grown by the Magnetron Sputtering system. Before sputtering process, the chamber was evacuated to a base pressure of 2.0 × 10^−7^ Torr. Afterward, the dielectric Si_3_N_4_ layer was deposited in various thicknesses in 30–250 nm range using a plasma-enhanced chemical vapor deposition (PECVD) system (PlasmaPro 800Plus, Oxford Instruments, UK). For the Si_3_N_4_ deposition, a gas mixture of SiH_4_, NH_3_, and N_2_ was used, and the substrate temperature was maintained at 250 °C. Our objective was to grow CrN pure thin film and fabricate it on a Si_3_N_4_ layer reactive magnetron sputtering system with a constant gas ratio of Ar (99.9999% pure) and N_2_ (99.9999% pure) to 10 sccm: 10 sccm. Before the sputtering process, the chamber was evacuated to a base pressure of 2.0 × 10^−7^ Torr, and 99.99% of ultra-high purity Cr target was used as the sputter target. The Auger Electron Spectroscopy (AES) data confirms that the 10 at. % of O_2_ is incorporated into the CrN system, and CrO_*x*_N_1−*x*_ was grown rather than pure CrN film. 400 W of DC sputtering power was introduced in each case of thin film deposition. The thickness of CrO_*x*_N_1−*x*_ film was tuned by controlling deposition time while other deposition conditions were kept constant. The deposition rate of CrO_*x*_N_1−*x*_ thin film was maintained at 0.63 nm/s. To measure the optical properties of the CrO_*x*_N_1−*x*_ thin film, samples of CrO_*x*_N_1−*x*_ thin film deposited on a transparent quartz substrate were also prepared using the same deposition conditions as those for the MIM samples. The cross-sectional microstructures of the tri-layer samples were analyzed by a transmission electron microscope (TEM) (FEI Talos F200X S/TEM, Thermo Fisher, USA). The cross-sectional TEM specimens were prepared using focused-ion-beam (FIB) milling after plating a thermoset epoxy on the top CrO_*x*_N_1−*x*_ layer. The reflectance spectra of the tri-layer samples were measured using a spectrophotometer (CM 3600A, Konica Minolta) equipped with a white xenon light source having a beam diameter of 4 mm; the detector and the incident light beam were positioned at 8° from the surface normal. The spectral refractive indices of the CrO_*x*_N_1−*x*_ layers fabricated with different *h*_m_ values were measured from those deposited directly on a quartz substrate using an ellipsometer (Elli-SEU-am12, Ellipsotech, South Korea). For comparison with the measured values, the reflectance spectra of the tri-layer samples were also simulated by solving the characteristic matrix using OpenFilters software [33], with inputs of the refractive indices and thicknesses of the individual layers. The pseudo-colors of the tri-layer samples were obtained from their reflectance spectra using Spectramagic NX software [34]. The absorption of each layer and phase shift were calculated by a trial version of the Semiconducting Thin Film Optics Simulation software (Setfos) S/W provided by Fluxim AG.

## 3 Calculation

**Figure 1:**
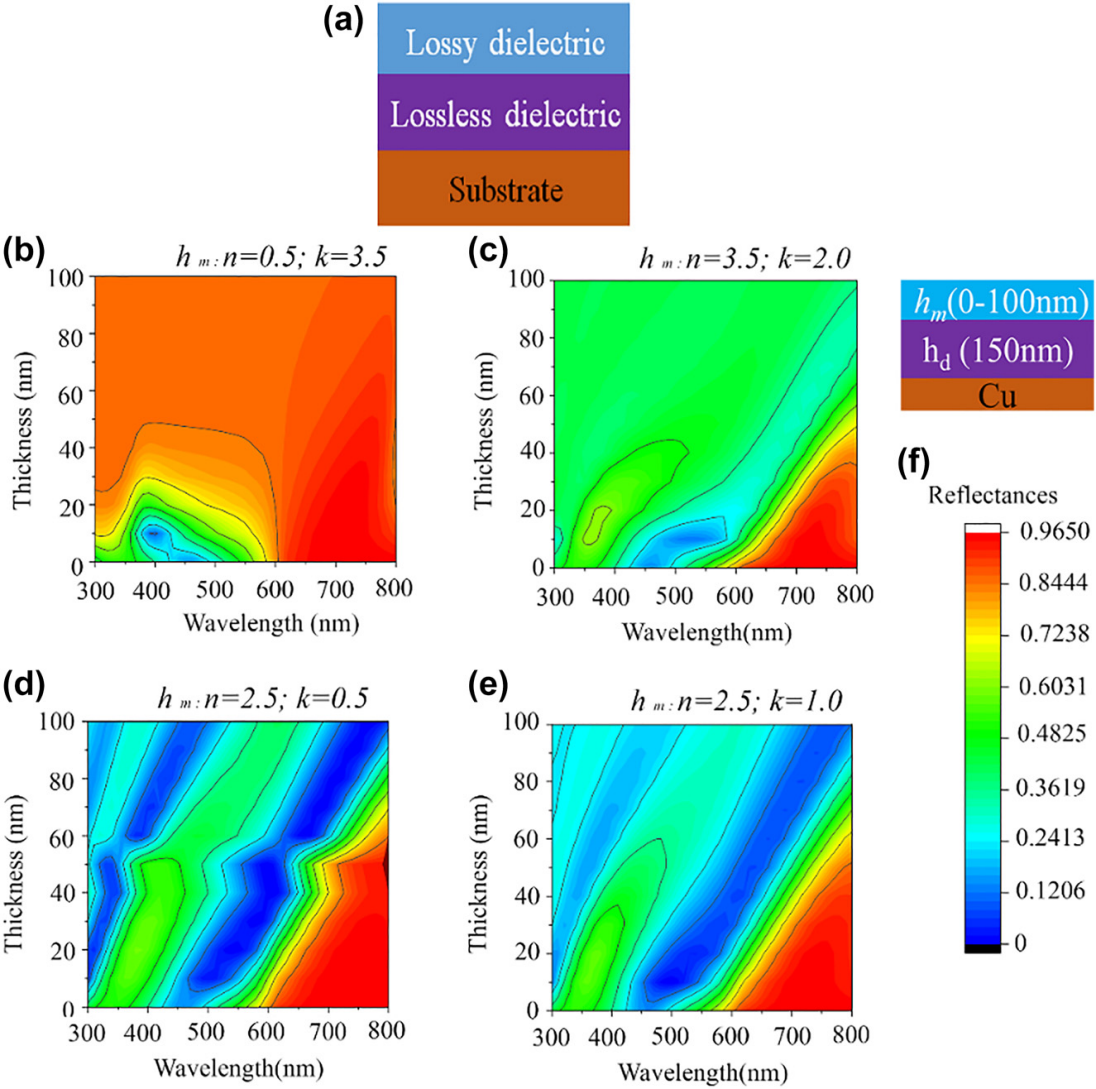
The calculated reflectance of the lossy dielectric-based trilayer structures as functions of the thickness of the lossy-dielectric-top layer and wavelength of the light. (a) The schematic of the structure. The dependence of the reflectance on the thickness of the top layer from 0 to 100 nm with refractive index of (b) *n* = 0.5; *k* = 3.5, (c) *n* = 3.5; *k* = 2.0, (d) *n* = 2.5; *k* = 0.5, (e) *n* = 2.5; *k* = 1.0. (f) The scale bar of reflectance.

In the present research, we propose a planar lossy-dielectric-based tri-layer design consisting of a lossy-dielectric on top, a lossless dielectric as intermediate, and an optically thick film can be used to generate reflective structural colors. The electric conductivity of electromagnetic waves in lossy metal σ_*m* is much greater than lossy dielectric σ_*l*, and for lossy dielectric, the electric conductivity is nearly equal to zero (σ_*l* = 0). Figure 1(a) illustrates a schematic diagram of such a design. A systematic simulation has been carried out to choose the top layer as a lossy dielectric through comparison with the reflectance of the MIM structure, which shows near-perfect absorption (>95%) could be realized for <80 nm-thick lossy dielectric films. As metals are highly lossy due to their high extinction coefficient values of *k* > 2.0, and therefore, as the thickness in the metal film increases to >40 nm, it acts like bulk metal, and most of the incident light is reflected from the top layer. In order to design a model structure for the realization of structural color by a thick top layer-based tri-layer cavity structure, the *k* value must be reduced to 0.5–1.0. A dependence of complex refractive indices on reflectance was calculated here to understand which refractive indices would be ideal for getting high absorption in the case of 40-nm-thick top layer-based tri-layer structure. To get rid of scratchiness and oxidation problem, Chromium Nitride (CrN) has been proposed, whose optical properties are very close to metal; *n* = 2.5–3.8, *k* = ∼2.0 over the visible range of wavelength (*λ*) as shown in Figure S1(a) (Supplementary Material). Chromium oxynitride (CrON) is excellent candidate for designing a thick tri-layer based F–P cavity structure due to its lower lossy property (*k* = 0.6–1.0) over the visible range of *λ* as shown in Figure S1(b) (Supplementary Material). Thus, highly saturated vivid structural colors could be generated for a thick CrO_*x*_N_1−*x*_ layer-based F–P cavity structure. Here, Si_3_N_4_ has been chosen for the intermediate layer, which is well known for the cavity layer. Furthermore, simulation and experimental works were carried out to generate reflective colors on copper, stainless steel, and aluminum substrates. The complex refractive indices of these metal substrates and the SiN_*x*_ are presented in Figure S2 (Supplementary Material).

Figure 1(b)–(e) illustrates a calculation of reflectance for four different types of top layer-based F–P cavity structures. Here, *h*_m_/*h*_d_ (150 nm)/Cu structure as shown in Figure 1(a) has been studied and reflectances were calculated for (b) *h*_m_: *n* = 0.5; *k* = 3.5*,* (c) *h*_m_: *n* = 3.5; *k* = 2.0, (d) *h*_m_: *n* = 2.0; *k* = 0.5, (e) *h*_m_: *n* = 2.5; *k* = 1.0. The dependences of top layer thickness were studied with varying *h*_m_ from 0 to 100 nm. The thickness (*h*_d_) of a lossless dielectric coating with a refractive index of 2.0 was set as 150 nm. For the convenience of calculation, the complex refractive index was considered constant over the visible range of *λ*. In most metals, e.g., Au, Ag, Cu, Cr, Ni, etc., the complex refractive indices show the relation of *n* < *k* over the visible range of *λ* since the substrate is Cu, for this case, the *T* (transmittance) = 0. Thus, *α* (absorption) = 1 − *R* (reflectance), which suggests that the lower the reflectance, the higher the absorption. Therefore, to understand the optical property of a metal-based tri-layer structure, we computed the reflectance of the generalized complex refractive index of 0.5 + 3.5*i* (*n* << *k*)*,* which represents the metallic structure-based F–P cavity structure **(** Figure 1(b)). The dependence of reflectance on the thickness of the top layer was studied, and it has been noticed that the thickness limit of the top layer is ∼10 nm to obtain strong light absorption (SLA). Figure 1(b) illustrates a very tiny strong blue region obtained at 400 nm wavelength for a 150 nm-thick cavity layer. The reflection of light is truly dominant over the visible range when increasing the top layer to *>*20 nm. To design a lossy-dielectric-based structure where *n* > *k,* a simulation was carried out for a top layer with a complex refractive index of 3.5 + 2.0*i* **(** Figure 1(c)). Here, the absorption region is slightly enhanced with the variation of top layer thickness for this kind of cavity structure but still limits the critical thickness of getting SLA at ∼10 nm. This behavior could be noticed while using Ge, CrN, etc., as the top layer of the tri-layer structure.

This work suggests an alternative strategy to achieve SLA (or even perfect light absorption (PLA)). We present that SLA can also be obtained when a tri-layer F–P cavity structure is designed with a top layer with a lossy-dielectric having comparatively lesser extinction coefficient (*n* >> *k*), and thus critical thickness of *h*_m_ to achieve SLA could be obtained at ∼100 nm. Figure 1(d) and (e) illustrates the dependences of reflectance with the F–P cavity structures with the complex refractive indices of the top layer of 2.5 + 0.5*i* and 2.5 + 1.0*i*, respectively. In both cases, SLA can be easily achieved for 10–100 nm thick lossy-dielectric-based tri-layer cavity structures. A shift in the resonance absorption towards the red wavelength region is noticed while increasing the *h*_m_ in the cavity structure for both cases (Figure 1(d) and (e)). Here, it is noted that Figure 1(b) corresponds to a typical lossy-metal, Figure 1(c) is a lossy-dielectric close to CrN, and (d) & (e) correspond to weak lossy dielectrics close to CrON.

**Figure 2: j_nanoph-2022-0522_fig_002:**
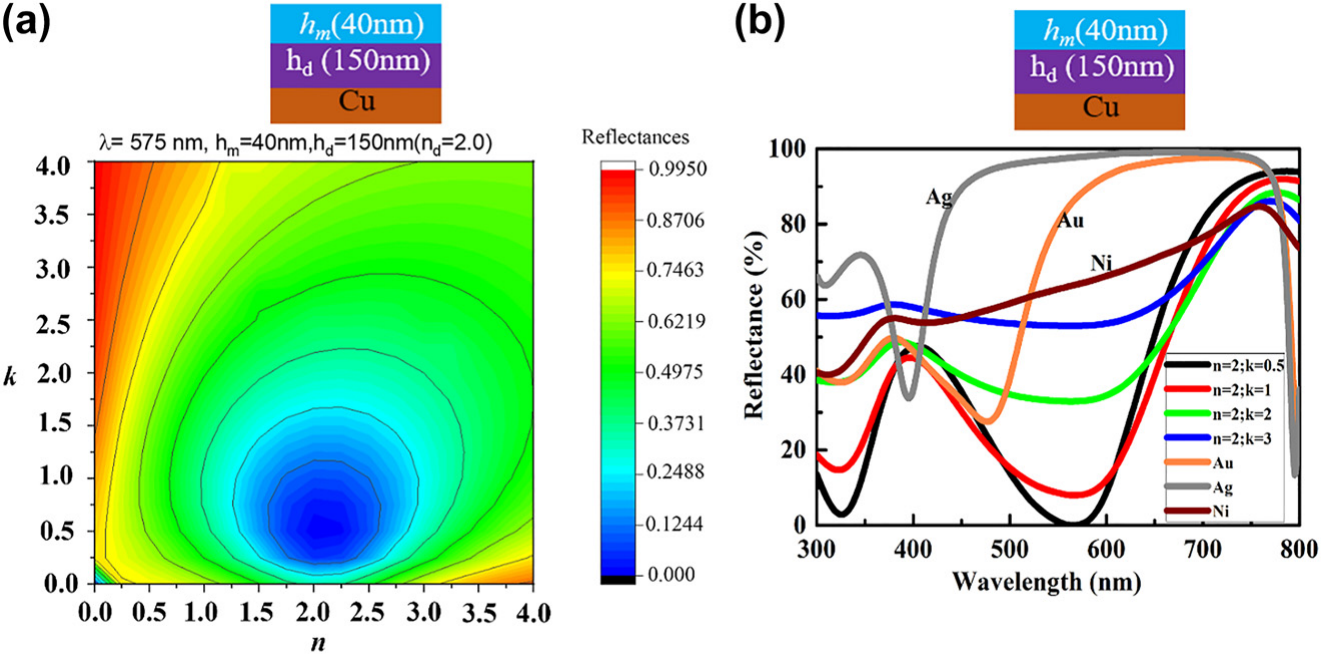
The dependences of the calculated reflectances and reflectance spectra with varying complex refractive indices for lossy dielectric based structures. (a) The dependences of the reflectance with varying *n* and *k* of the suggested structure are shown as schematic. (b) The dependences of the reflectance spectra with varying wavelength for the tri-layer structure having lossy dielectric on top (*n* = 2, *k* = 0.5; *n* = 2, *k* = 1.0) compared with lossy metals with refractive indices of *n* = 2, *k* = 2; *n* = 2, *k* = 3 and Au, Ag, and Ni.

In order to make a model of planar-design, a systematic calculation is required to understand which complex refractive indices are best suitable for the thick top-layer based tri-layer F–P cavity structure. Figure 2(a) illustrates the dependence of complex refractive indices on reflectances for a thick top-layer (*h*_m_ = 40 nm) based tri-layer structure with a cavity thickness of 150 nm on a reflective Cu substrate. The reflectance was calculated and presented here for a constant *λ* of 575 nm. As seen from Figure 2(a), SLA can be achieved with the complex refractive indices of *n* = 1.8–2.5 and *k* = 0.5–1.0. A full wavelength (300–800 nm) reflectance spectra were calculated with variation of *n* and *k* values and two classes are presented: (a) lowly absorptive material where *n* = 2, *k* = 0.5–1 and (b) moderately higher with equal or near values of *n* and *k* values (*n* = 2, *k* = 2–3). These reflectances calculated with these refractive indices were also compared with the same structure where Au, Ag, and Ni metal layers were considered.

As we see from the calculated spectra, a thick layer (40 nm in this case) with the lowly absorptive material with *n* of 2.0 and *k* of 0.5–1.0 can be easily generate SLA while all other case exhibits high reflection (Abs. ≤70% at resonance absorption).

**Figure 3: j_nanoph-2022-0522_fig_003:**
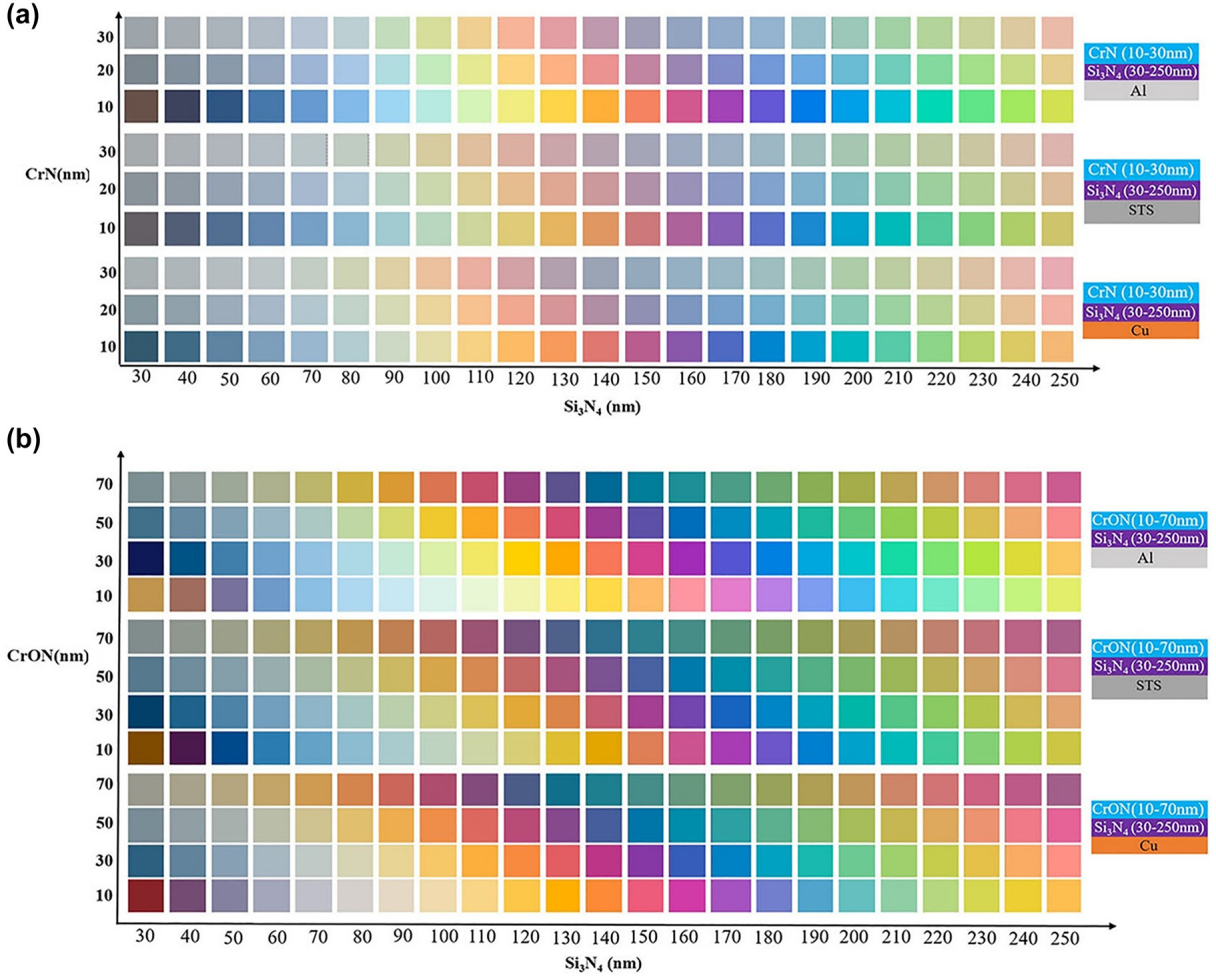
Predicted structural colors of the (a) CrON/Si_3_N_4_/Al, CrON/Si_3_N_4_/STS and CrON/Si_3_N_4_/Cu structures and (b) CrN/Si_3_N_4_/Al, CrN/Si_3_N_4_/STS and CrN/Si_3_N_4_/Cu.

Bulk CrN is known for its hardness and corrosion resistive properties. PVD-coated CrN provides excellent wear and corrosion resistance [32]. Inspired from the physical properties of CrN, structural color was predicted for a triple layer, CrN (*h*_m_)/Si_3_N_4_ (*h*_d_)/substrate and these data were compared with CrON (*h*_m_)/Si_3_N_4_ (*h*_d_)/substrate structure. For the calculation all three substrates Al, STS, Cu were considered. Figure 3(a) illustrates the calculated colors of CrN (*h*_m_)/Si_3_N_4_ (*h*_d_)/Al, CrN (*h*_m_)/Si_3_N_4_ (*h*_d_)/STS and CrN (*h*_m_)/Si_3_N_4_ (*h*_d_)/Cu structures. Thickness of CrN layer was tuned from 10–30 nm and the color realized from CrN top layer is very close to the reported metallic top layer-based colors, where thinner layer of 10 nm provides best color purity and vividness. As thickness of CrN top layer was increased, these structures’ color quality degraded regardless of the substrates’ nature. The calculated colors of CrON (*h*_m_)/Si_3_N_4_ (*h*_d_)/Al, CrON (*h*_m_)/Si_3_N_4_ (*h*_d_)/STS and CrON (*h*_m_)/Si_3_N_4_ (*h*_d_)/Cu structures were illustrated in Figure 3b. The CrON top layer with the thickness range of 10–70 nm was used for the calculation of colors and realization of vivid colors are realized by a broader range of structures, regardless of the substrates are used, i.e. for Al, STS, and Cu. The overall color quality of the CrON-top layer-based structures is much better than CrN- the top-layer-based F–P cavity structure. Both *h*_m_ and *h*_d_ can be used to tune the colors for CrON-based structure and a red shift in colors is noticed as they are increased in both cases. The red shift noticed due to the variation of the top layer thickness suggests a possible interference effect occurred in the CrON layer.

**Figure 4: j_nanoph-2022-0522_fig_004:**
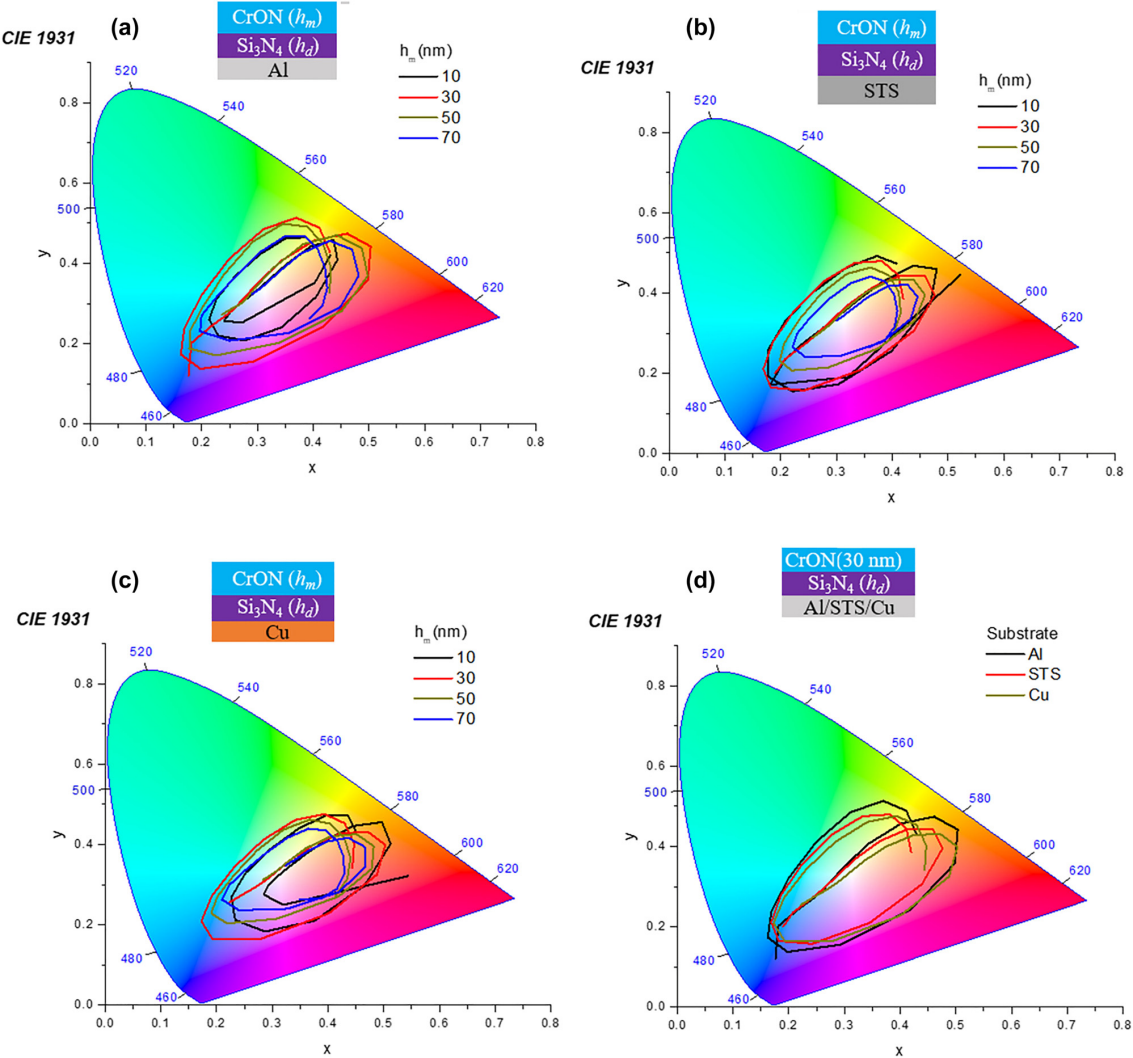
CIE 1931 chromaticity diagram illustrating the CIE coordinates of the simulated (a) CrON/Si_3_N_4_/Al, (b) CrON/Si_3_N_4_/STS, and (c) CrON/Si_3_N_4_/Cu structures as a function of *h*_m_ and *h*_d_; *h*_m_ = 10–70 nm and *h*_d_ = 30–250 nm.

All the simulated colors of CrON top layer based structures for Al, STS and Cu are shown in Figure 3(b), are mapped in the International Commission on Illumination (CIE) 1931 chromaticity diagram and depicted in Figure 4(a)–(c). Highest color saturation for these structures was noted for 30-nm-thick CrON- top layer-based structures on all three substrates. The CIE color coordinates of CrN top layer-based structures are presented in Figure S3 (Supplementary Material), high-quality structural colors could only be realized for CrN 10 layer-based-structures regardless of the type of the substrates, as thickness of CrN layer was increased weakly saturated colors were appeared.

To understand the effect of colors on various substrates, the CIE chromaticity parameter was plotted for 30 nm-thick CrON- top layer-based structures for these substrates, as illustrated in Figure 4(d). The overall color saturation for Al substrates is higher than STS and Cu. The color saturation was also considerably well for the Cu substrates, which has its intrinsic colors as well gray colored substrates Al, and STS which provides broader range of industrial application.

**Figure 5: j_nanoph-2022-0522_fig_005:**
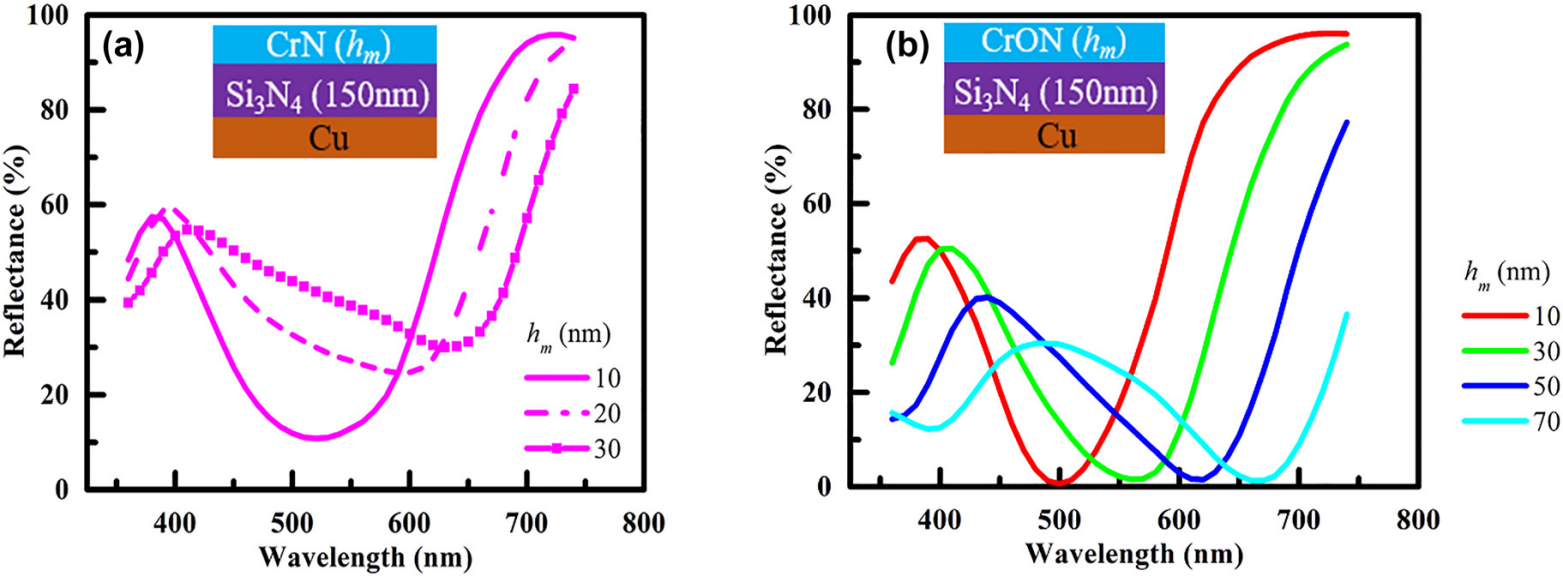
The reflectance spectra of (a) CrN (*h*_m_)/Si_3_N_4_/Cu structures varying *h*_m_ from 10 to 30 nm; as the thickness of CrN increases, the absorption reduces significantly. (b) The reflectance spectra of CrON (*h*_m_)/Si_3_N_4_/Cu structures varying *h*_m_ from 10 to 70 nm; SLA can be noticed in all four cases and no significant reduction of absorption is detected as the thickness of CrON layer was increased.

The reflectance spectra of CrN (*h*_m_)/Si_3_N_4_/Cu and CrON (*h*_m_)/Si_3_N_4_/Cu structures are illustrated on Figure 5(a) and (b), respectively. Here, the thickness of Si_3_N_4_ was considered as 150 nm in both cases, and *h*_m_ was 10–30 nm (step = 10 nm) and 10–70 nm (step = 20 nm). Wide-band absorption is noticed at *λ* of = 520–540 nm for CrN (*h*_m_ = 10 nm) and as the thickness increases, resonance absorption shows a red shift; *λ*_res_ of 580–600 nm for CrN (*h*_m_ = 20 nm) and *λ*_res_ of 630–650 nm for CrN (*h*_m_ = 30 nm). The absorption value reduces linearly as the CrN thickness increases and the CrN (*h*_m_ = 10 nm) layered structure exhibits the best absorption behavior with a value of 90%. In contrast to the CrN-based structure, CrON (*h*_m_)/Si_3_N_4_ (150 nm)/Cu structures exhibit narrow band absorption in the resonance wavelength. The resonance absorption in the CrON case shows a similar red shift as seen in the CrN-based structure, but absorption value remains constant as the thickness of the CrON layer increases. PLA (100% abs.) could be achieved by the four structures presented in Figure 5(b). As the thickness is increased from 10 to 30, 50 and 70 nm, *λ*_res_ appears at 525, 580, 650 and 690 nm, respectively. The refractive indices of CrN and CrON used to calculate the reflectance and color of the tri-layer structures are shown in Figure S2 (Supplementary Material). Here, previously reported refractive indices of CrN were used [35], and optical properties of CrON were obtained from the ellipsometry measurement.

The complex refractive index of CrN is *n* of 2.5–3.8 and *k* of ∼2.0, whereas the complex refractive index of CrON is *n* of ∼2.0 and *k* of 0.6–1 over the visible wavelength range. Thus, the absorption of the CrN layer is moderately higher than that of the CrON when the two materials have the same thickness. However, the reflection of the incident light wave at the air-CrN interface is higher than the reflection of the incident wave at the air-CrON interface because the real part of the complex refractive index of CrN is larger than the real part of the CrON complex refractive index. At the interface with refractive indices *n*_1_ and *n*_2_, the interface reflection of a light wave that is usually incident is given by *R* = {(*n*_1_ − *n*_2_)/(*n*_1_ + *n*_2_)}^2^. In the lossy-dielectric-based tri-layer structures, as schematically shown in Figure S4(a) (Supplementary Material), the resonance region broadens, and the resonance intensity decreases according to Δ, the difference between *h*_m_ and *h*_d_. Thus, as the thickness of *h*_m_ increases, the resonance region broadens, and the resonance intensity decreases. Also, as the *h*_m_ increases, the peak resonance positions for both CrN and CrON are red-shifted, and the bandwidth (line width) increases simultaneously. Meanwhile, in the MIM structure (Figure S4(b)) (Supplementary Material), the resonance peak changes only according to the thickness of the insulating layer (lossless dielectric layer). Since Δ is constant, the bandwidth does not change significantly compared to the suggested structure.

**Figure 6:**
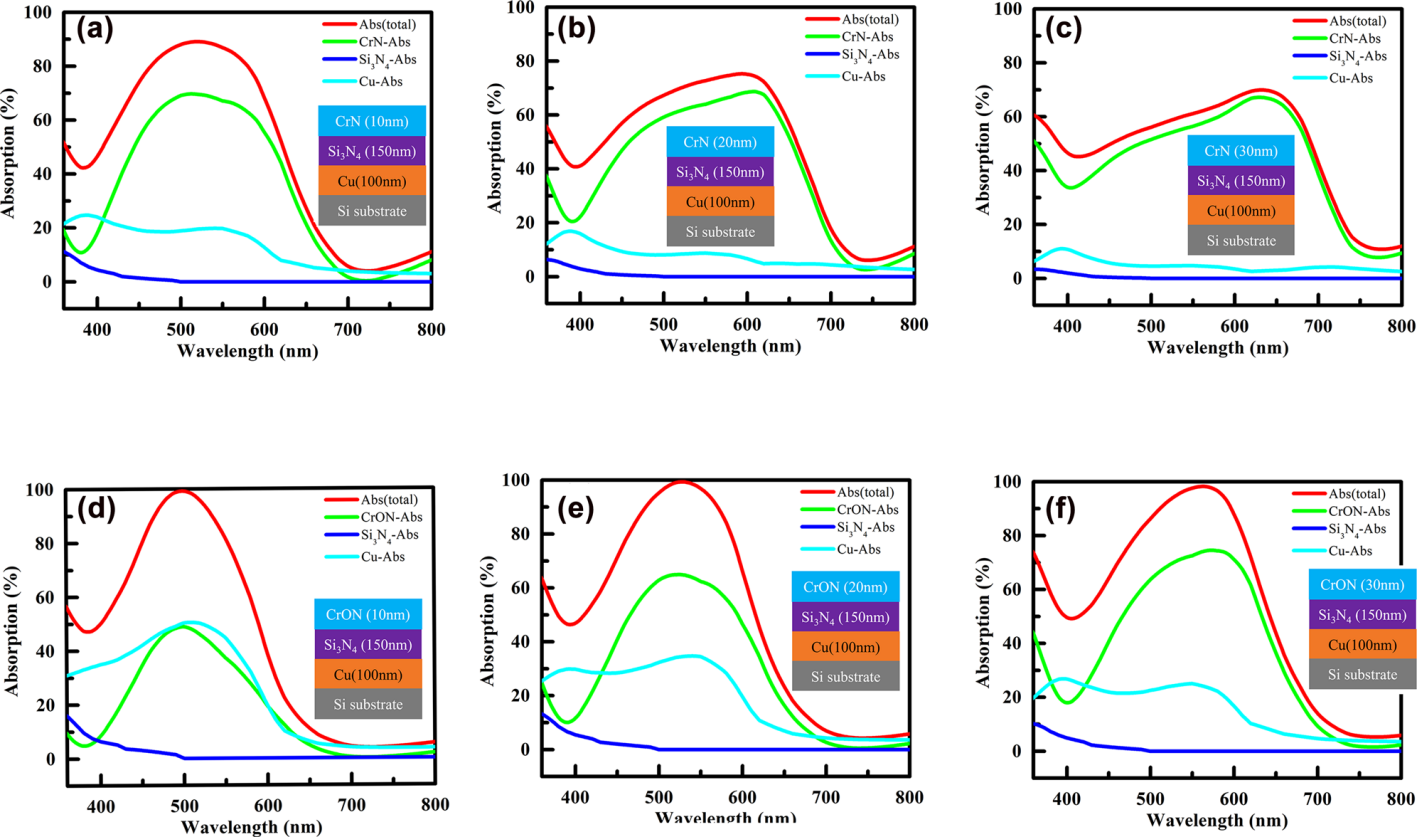
The calculated absorption of each layer for: (a)–(c) the CrN-based structures and for: (e)–(f) the CrON-based structures.

In order to understand the effect of each layer for the realization of structural color in CrN top layer and CrON-top layer-based structures, absorption of the total fractions of the incident light within each layer of the CrN (10–30) nm/Si_3_N_4_ (150 nm)/Cu (100 nm)/Si and CrON (10–30) nm/Si_3_N_4_ (150 nm)/Cu (100 nm)/Si structures are computed and illustrated in Figure 6(a)–(f), respectively. Wide band absorption is noticed in the CrN based structure, while the CrON-based-structures present narrow band absorption. A significant value-drop in the total absorption can be clearly seen from the CrN-based structures as the CrN thickness increases from 10 to 30 nm. That is, the CrN (10 nm)-based structure exhibits a maximum absorption value of 89% (at 518 nm), while the maximum absorption drops to 75% (at 594 nm) and ∼70% (at 632 nm) for CrN (20 nm)- and CrN (30 nm)-based structures, respectively. In the case of the CrN-based structure, it is noticed that most of the absorption occurs in the CrN layer as opposed to the bottom Cu layer. Herein, the 10 nm CrN sample provides the total absorption of 89%, and the rest of 11% of the light gets reflected. Here ∼70% of the incident light is absorbed by the CrN layer, where ∼19% of the light is absorbed in the Cu layer. The contribution of the Cu layer in absorption is primarily reduced when the thickness of CrN layer is increased to 20, and 30 nm, and absorption in the Cu layer turns to 7 and 3%, respectively, stating that most of the lights are absorbed in the CrN layer. On the other hand, the CrON-based structures exhibit 99% absorption (98% for 30 nm-thick CrON sample), a significant contribution of the absorption comes from both the Cu bottom layer and the CrON layer. As the CrON thickness increases, the absorption rate of Cu layer reduces while that of CrON increases. The trend of absorption-reduction in the Cu layer observed in the CrON samples is similar to the trend observed in the CrN case. Still, even after this reduction, the CrON (30 nm) based structures exhibits 24% of absorption of light by the Cu layer (the remaining 74% of light is absorbed in the CrON layer, and 1% of the light gets reflected). The reflectance spectra were also calculated for the CrN (100 nm)–Si_3_N_4_ (*h*_d_)–Cu (100 nm) structures and reflectance spectra varying wavelength are shown in Figure S5 (also see Table S1). It can be seen that >∼90% absorption can be achieved in CrN (100 nm) based structure, even though overall reflectance is lower than thinner CrN-based structures (Figure 5). The effect of incident angle on the reflectance spectra varying wavelength for both thinner CrN- and thicker CrN-based structures were studied and both exhibit a similar trend of negligible shift in reflectance spectra for 10-degree incident angle, with no significant change in *R*-min value. As the incident angle increases to 30°, absorption wavelength *λ* (abs) exhibits a blue shift of 15 nm for CrN (30 nm)/Si_3_N_4_ (150 nm)/Cu structures and additional 15 nm blue shift occurs for 50° incident angle. The *R*-min value remains in the range of 1.5–6.95. A similar trend of blue shift, 13 and 26 nm blue-shift for 30 and 50° incident angle, respectively, is noticed for CrN (100 nm)/Si_3_N_4_ (90 nm)/Cu structures. The *R*-min value remains in the range of 4.50–10.95 for CrN (100 nm)/Si_3_N_4_ (90 nm)/Cu structures varying incident angle from 0 to 50° (Figure S6, Tables S2 and S3).

To explain the observed results, we consider the optical effect by the difference in the refractive indices between the adjacent layers in the suggested structures. The reflection of the incident wave at the air-CrN interface is higher than the reflection of the incident wave at the air-CrON interface. Also, the absorption of CrN-layer becomes larger than that of CrON-layer for the same thickness since the absorption coefficient of CrN (imaginary part of complex refractive index) is higher than that of CrON. Moreover, as the thickness of CrN-layer increases, the overall absorption of CrN/Si_3_N_4_/Cu structure decreases, while that of CrON/Si_3_N_4_/Cu structure is nearly the same as the thickness of CrON increases. In the case of CrN, the amount of light incident into the F–P structure is reduced as the thickness of CrN increases. Also, since the refractive index difference between the CrN (∼3.5 + 2*i*) and the Si_3_N_4_ (∼2 + 0*i*) is slightly larger than that between the Si_3_N_4_ (∼2 + 0*i*) and the Cu (∼0.8 + 3*i*), the degree of internal multiple reflection at the CrN–Si_3_N_4_ boundary is slightly greater than at the SiN_*x*_–Cu boundary. Therefore, the change in the absorption rate on the Cu side is affected more, and the total absorption spectra decreases as the thickness of CrN increases, as shown in Figure 6(a)–(c). Meanwhile, since the refractive index difference (∼1.2) between the Si_3_N_4_ and the Cu substrate is larger than that (∼0.1) between the CrON and the Si_3_N_4_, the effect of internal multiple reflection at the Si_3_N_4_–Cu boundary is larger than that at the CrON–Si_3_N_4_ boundary, resulting in the increased absorption in the CrON side. Therefore, in this structure, the overall absorption is thought to be maintained high (>98%) due to the complementary effect of CrON and Cu absorption. That is, as the thickness of CrON increases, the absorption in CrON increases and the absorption in Cu decrease, as shown in Figure 6(d)–(f).

**Figure 7:**
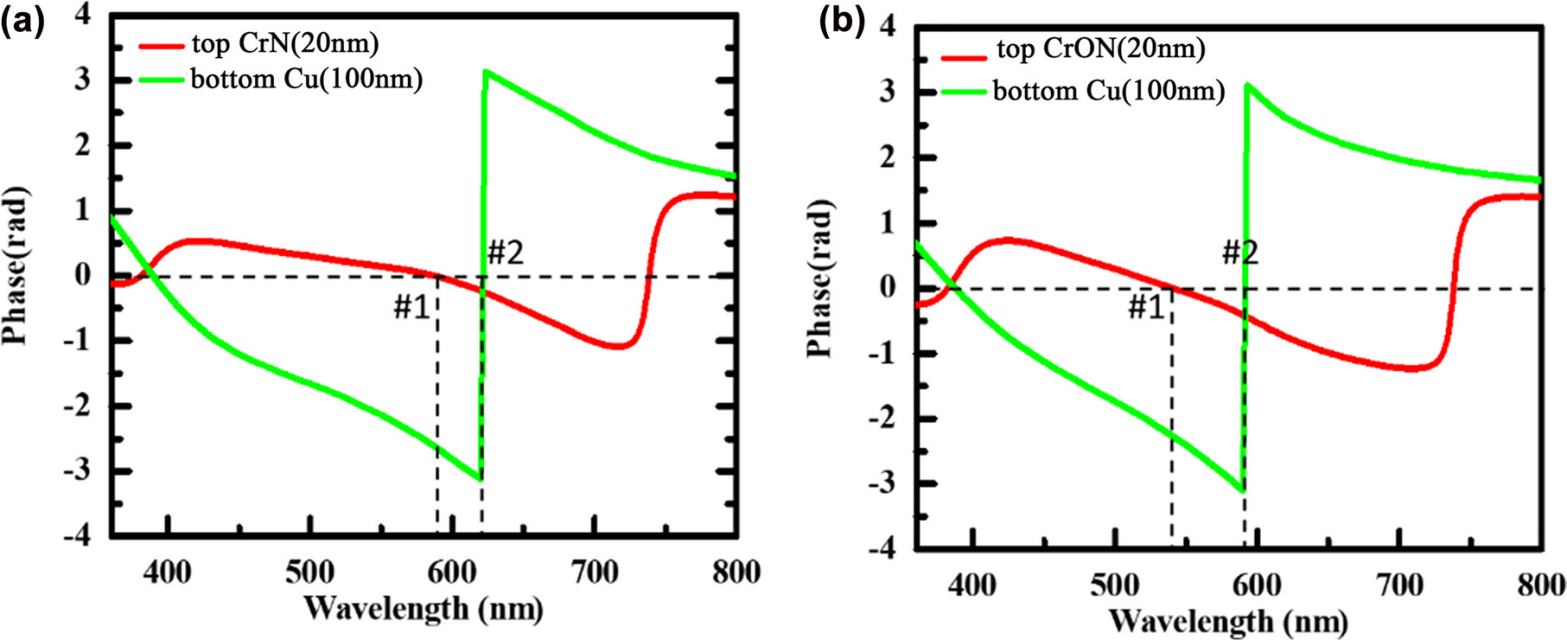
The calculated net phase shift in the top CrN (20 nm) and bottom Cu layers of (a) the CrN (20 nm)/Si_3_N_4_ (150 nm)/Cu (100 nm) structures, and the calculated net phase shift in the top CrON (20 nm) and bottom Cu layers of (a, b) the CrON (20 nm)/Si_3_N_4_ (150 nm)/Cu (100 nm) structures.

In order to investigate the resonance absorption more deeply, the net phase shift of the top and bottom layers in CrN and CrON-based structures were calculated. The net phase shift illustrated here involves the reflection phases occurred at both the top and bottom interfaces and the accumulation of the propagation phases within the Si_3_N_4_ layer for the CrN- and CrON-based structures. The red-colored spectra of Figure 7(a) and (b) illustrate the calculated net phase shift in the top CrN (20 nm) and CrON (20 nm) layer of the CrON (20 nm)/Si_3_N_4_ (150 nm)/Cu (100 nm) and CrON (20 nm)/Si_3_N_4_ (150 nm)/Cu (100 nm) structures, respectively. Absorption resonances shown in Figure 6 appear when the net phase shift is equal to 0. It can be seen that the resonance modes are obtained in the top CrN and CrON cavities at 587 (#1) and 540 nm (#1), respectively, which are very close to the absorption value obtained for these two structures (Figure 6). The green-colored spectra of Figure 7(a) and (b) illustrate the calculated net phase shift in Cu (100 nm) reflector in the CrON (20 nm)/Si_3_N_4_ (150 nm)/Cu (100 nm) and CrON (20 nm)/Si_3_N_4_ (150 nm)/Cu (100 nm) structures, respectively and #2 represents the absorption contribution in Cu layer.

The refractive indices of bulk Cu are presented in Figure S2(a) (Supplementary Material), which are used to calculate reflectance and colors shown in Figures 1 [Fig j_nanoph-2022-0522_fig_002] [Fig j_nanoph-2022-0522_fig_003] [Fig j_nanoph-2022-0522_fig_004] [Fig j_nanoph-2022-0522_fig_005]–6.

Thin films having anisotropic properties are formed in an asymmetric pattern exhibit optical properties that change depending on the polarization direction (TE or TM) of incident light. For example, the highly asymmetric SPP (surface plasmon polariton) effect due to the anisotropy of black phosphorus (BP) allows a BP-SiC metasurface to be used as anisotropic absorber and tunable source of MIR radiation [36]. Also, an asymmetric THz metasurface formed by displacing two adjacent metal arms exhibits a polarization-dependent electromagnetic response [37]. On the other hand, the CrON thin films having uniform and isotropic properties [38] reveals characteristics independent of the polarization direction of incident light as shown in Figure S7.

## 4 Results and discussions

**Figure 8:**
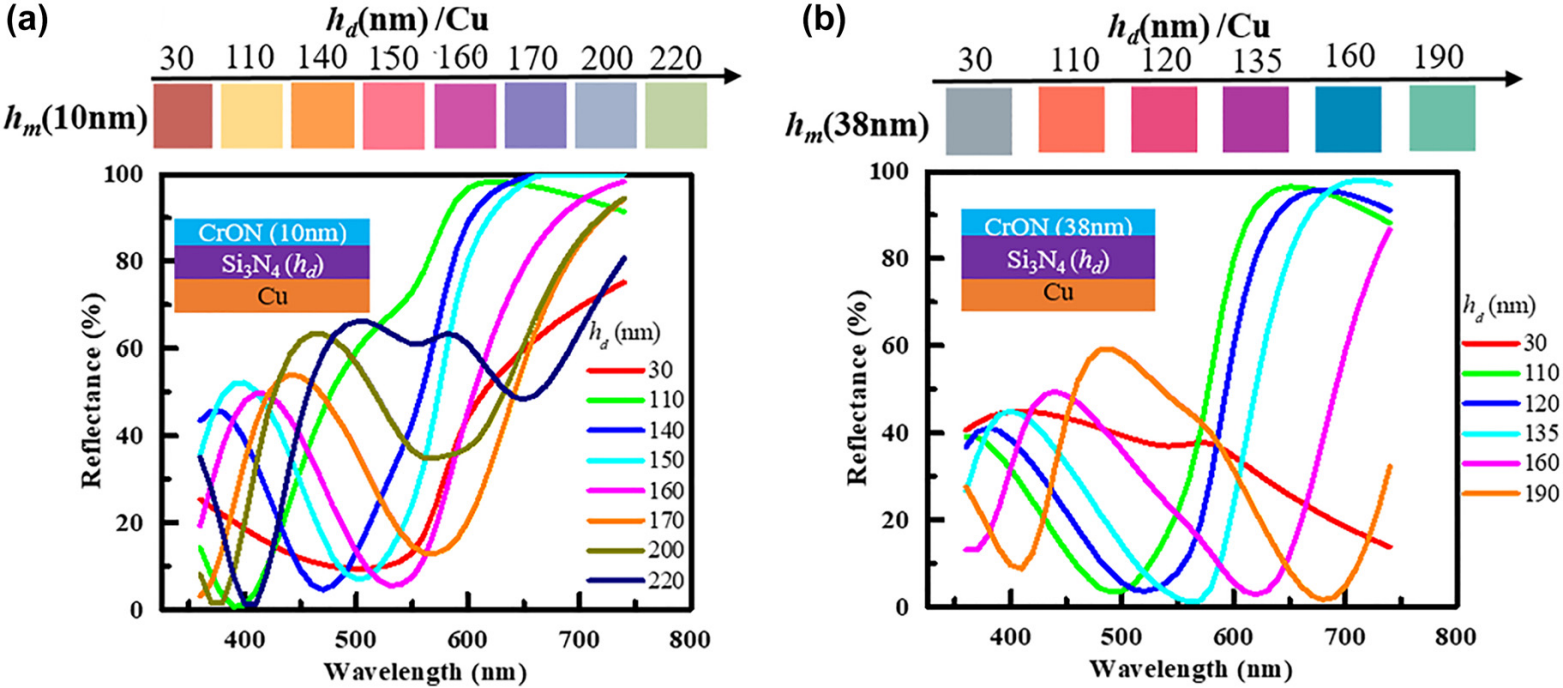
The measured reflectance spectra and corresponding colors of the fabricated (a) CrON(10nm) /Si_3_N_4_(*h_d_*)/Cu and (b) CrON (38nm)/Si_3_N_4_(*h_d_*)/Cu structures.

Figure 8 shows the experimentally observed colors along with the reflectance spectra of the CrO_*x*_N_1−*x*_ (10 nm)/Si_3_N_4_ (*h*_d_)/Cu (100 nm)/Si and CrO_*x*_N_1−*x*_ (38 nm)/Si_3_N_4_ (*h*_d_)/Cu (100 nm)/Si structure. For these CrOxN_1−*x*_ (10 nm)- and CrOxN_1−*x*_ (38 nm)-based structures, the variation in the thickness of the lossless cavity layer and/or the lossy CrO_*x*_N_1−*x*_ layer leads to a range of colors including pink, violet, magenta and blue colors. Resonance absorption shows a red shift as the cavity thickness increases in the CrO_*x*_N_1−*x*_-based structures, which reveals the resonance cavity mode [15].

**Figure 9:**
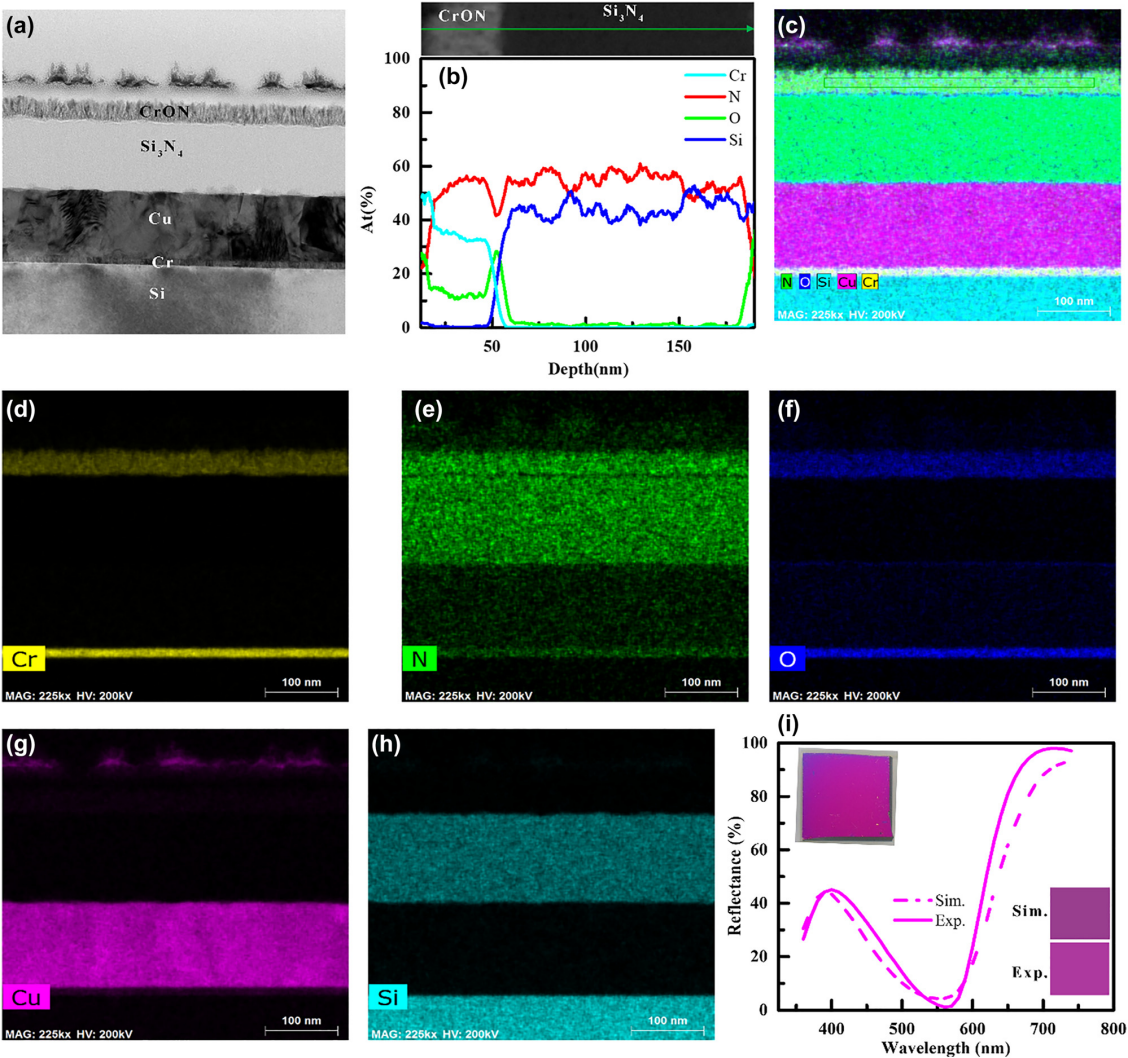
The cross-sectional transmission electron microscopy (TEM) images, elemental line profile and Energy Dispersive Spectroscopy (EDS) mapping images of the CrO_*x*_N_1−*x*_ (38 nm)/Si_3_N_4_ (135 nm)/Cu (130 nm)/Si structure. (i) The simulation spectrum and estimated color (bottom inset) are compared with the experimental spectrum and observed color (bottom inset). Top inset shows the camera images of the color produced by the fabricated sample.

The cross-sectional transmission electron microscopy (TEM) images, elemental line profile and Energy Dispersive Spectroscopy (EDS) mapping images of CrO_*x*_N_1−*x*_ (38 nm)/Si_3_N_4_ (135 nm)/Cu (130 nm)/Si structure, are presented in Figure 9(a)–(h). Elemental line profile and EDS mapping images confirm each element of the structure. A 13 nm-thick Cr layer was used as an adhesion layer on a Si substrate. The thickness of each layer of the TEM specimen was estimated and the thickness corrected for simulation has been carried out for the specimen, illustrated in Figure 9(i). The measured refractive indices of the Cu thin film (Figure S2(b)) (Supplementary Material) were used to calculate the reflectance and the color. The spectrum and colors obtained from the simulation reasonably match well with the experimental counterparts. The inset showing the specimen’s camera images also validate the color.

**Figure 10:**
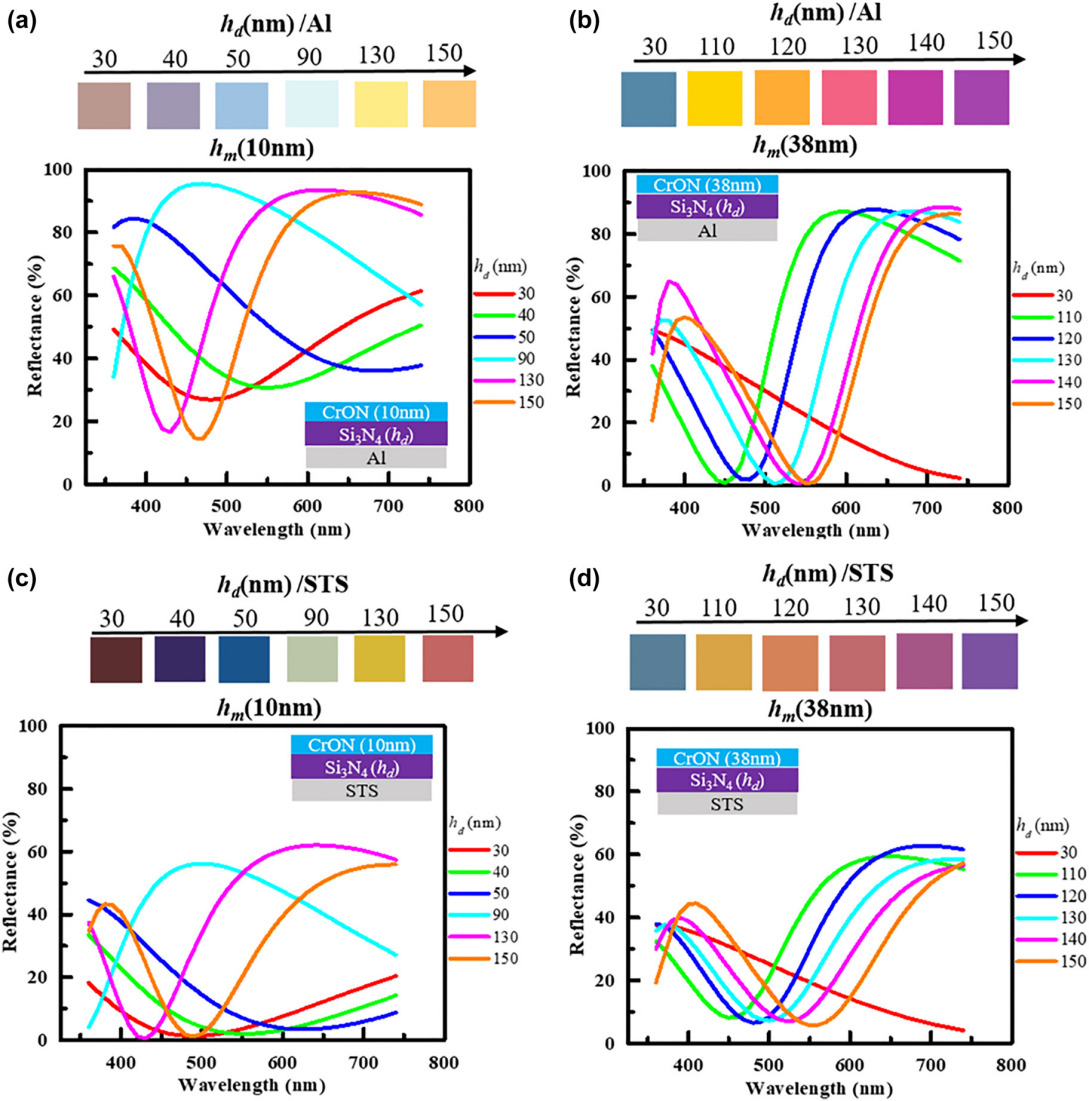
The measured reflectance spectra and corresponding colors of the fabricated (a) CrON (10 nm)/Si_3_N_4_ (*h*_d_)/Al, (b) CrON (38 nm)/Si_3_N_4_ (*h*_d_)/Al, (c) CrON (10 nm)/Si_3_N_4_ (*h*_d_)/STS, and (d) CrON (38 nm)/Si_3_N_4_ (*h*_d_)/STS structures.

Using the CrO_*x*_N_1−*x*_-based tri-layer structures, the experimental colors were also fabricated on aluminum (Al) and stainless steel (STS) back reflectors. Figure 10 provides the reflectance spectra and color of the CrON/Si_3_N_4_ structure deposited on the Al and STS substrates.Figure 10(a) shows the reflection spectra and color of the cavity samples of the CrO_*x*_N_1−*x*_ (10 nm)/Si3N4 (*h*_d_)/Al structures, fabricated with *h*_d_ = 30, 40, 50, 90, 130 and 150 nm, and various reflection colors, i.e. brown, violet, cyan, cyan-green, yellow and orange can be realized, respectively. Figure 10(b) illustrates the reflection spectra and color of the cavity samples of the CrO_*x*_N_1−*x*_ (34 nm)/Si_3_N_4_ (*h*_d_)/Al structures, fabricated with *h*_d_ = 30, 110, 120, 130, 140 and 150 nm, and various reflection colors, i.e. cyan, yellow, light-orange, pink, magenta and violet can be realized, respectively. The overall color vividness increases as the absorption in the reflection dip increases for the CrON based structures fabricated on Al substrate. Here, SLA can be also seen in the thicker CrO_*x*_N_1−*x*_-based structures when the Al reflector was considered. As the thickness of CrON increases, there is a similar tendency in reflectance spectra to the case of the Cu substrate (the thicker the CrON thickness, the higher the absorption in CrON). From the complex refractive indices of the metals and lossy dielectrics over the visible wavelength range as seen in the Figures S1 and S2, the difference (∼0.5) in the real part of the complex refractive index of Si_3_N_4_–Al boundary is higher than the difference (∼0.1) in the real part of the complex refractive index of CrON–Si_3_N_4_. Thus, the multiple reflection at the SiN_*x*_–Al interface is still larger than that at the CrON–SiN_*x*_ interface. The increased absorption as the thickness of CrON increases greatly affects the total absorption. In case of the reflectance spectra of CrON/Si_3_N_4_ on STS substrate as shown in Figure 10(c) and (d), the thickness of CrON correspond to 10 and 38 nm. A range of vivid colors could be realized on the STS surface by tuning the thickness of the lossy and lossless dielectric. A spectrum of colors realized in this case including violet, blue, magenta and yellow. In case of STS reflector, SLA is noticed in the thinner CrO_*x*_N_1−*x*_ (10 nm)-based structure and color vividness slightly decreases due to slight deterioration of absorption value (decrease abs. % from ∼100% to 92–95%), as the thickness of CrO_*x*_N_1−*x*_ increases. The absorption tends to decrease slightly as the CrON thickness increases. This is a situation in which the difference in the real part of the complex refractive index of CrON–Si_3_N_4_ is more significant than the difference in the real part of the complex refractive index of Si_3_N_4_–STS. Thus, the multiple reflection in this F–P structure is higher at the CrON–Si_3_N_4_ boundary, meaning that the degree of absorption at the STS side is affected more. As the CrON thickness increases, the absorption of the metal substrate tends to decrease. When a colored substrate such as Cu is used as back reflector of F–P cavity structure, the reflectance dip or peak appears asymmetry, which is attributed to the mixing the original color of the substrate and the F–P interference color of the trilayer. Meanwhile, when a substrate such as Al or STS is used, the reflectance dip or peak shows symmetry due to no color mixing by the substrate with the F–P interference color.

The present study provides sufficient information about an asymmetric F–P cavity structure where top-layer was considered as lossy-dielectric. This structure can be used to realize reflective colors on various substrates. The realized colors are due to a combination of interference effect of asymmetric F–P cavity structure and the absorption rate in the CrO_*x*_N_1−*x*_ layer.

## 5 Conclusions

A planar asymmetric F–P cavity structure consisting of a lossy-dielectric as top-layer, is proposed for the structural coloration which can be perfect replacement for the metal top-layer-based MIM structure. Due to its inherent corrosion and wear resistive property CrN is a very candidate to realize structure based on this scheme. A refractive index dependency on the reflectance data provides that CrON could be a good fit as the top lossy-dielectric. The simulation data of the CrN- and CrON-based structure was compared, and it was noticed the CrON-based structure is more promising as *h*_m_ ≥ 100 nm can provide SLA, hence produces vivid structural colors. The CrO_*x*_N_1−*x*_-based tri-layer structures were fabricated on the Cu, Al, and STS back reflectors and the spectra of vivid colors can be generated. The colors can exhibit a red shift when the Si_3_N_4_ cavity layer is increased. The colors also show a red shift when the thickness of the CrO_*x*_N_1−*x*_ layer increases. This suggests that the interference occurred in the asymmetric F-P cavity as well as the absorption rate of the CrO_*x*_N_1−*x*_ layer led to produce vivid colors. This scheme presented here is very simple and therefore, could be very useful for the industrial application such as perfect absorber, surface decoration, art and optical filter.

## Supplementary Material

Supplementary Material Details
