# Supplementary material for: To realize a variety of structural color adjustments via lossy-dielectric-based Fabry–Perot cavity structure
Source: Nanophotonics. 2022 Nov 3;11(21):4855–68. doi: 10.1515/nanoph-2022-0522 (PMC11502086; doi:10.1515/nanoph-2022-0522)
Supplement: Supplementary file 1 — Supplementary Material Details [file j_nanoph-2022-0522_suppl.docx]

Supporting Information

**To realize a variety of structural color adjustments via lossy-dielectric-based Fabry–Perot cavity structure**

M.A. Rahman, ^a^ Dong Kyu Kim,^a^ Jong-Kwon Lee,^b^ and Ji Young Byun*, ^a^

^a^Extreme Materials Research Center, Korea Institute of Science & Technology, 5, Hwarang-ro 14-gil, Seongbuk-gu, Seoul 02792, Republic of Korea

^b^Division of Energy and Optical Technology Convergence, Cheongju University, Cheongju-si Chungcheongbuk-do 28503, Republic of Korea

* Corresponding author at: Extreme Materials Research Center, Korea Institute of Science & Technology,

5, Hwarang-ro 14-gil, Seongbuk-gu, Seoul 02792, Republic of Korea.

E-mail address: [jybyun@kist.re.kr](mailto:jybyun@kist.re.kr) (J.Y. Byun).

**
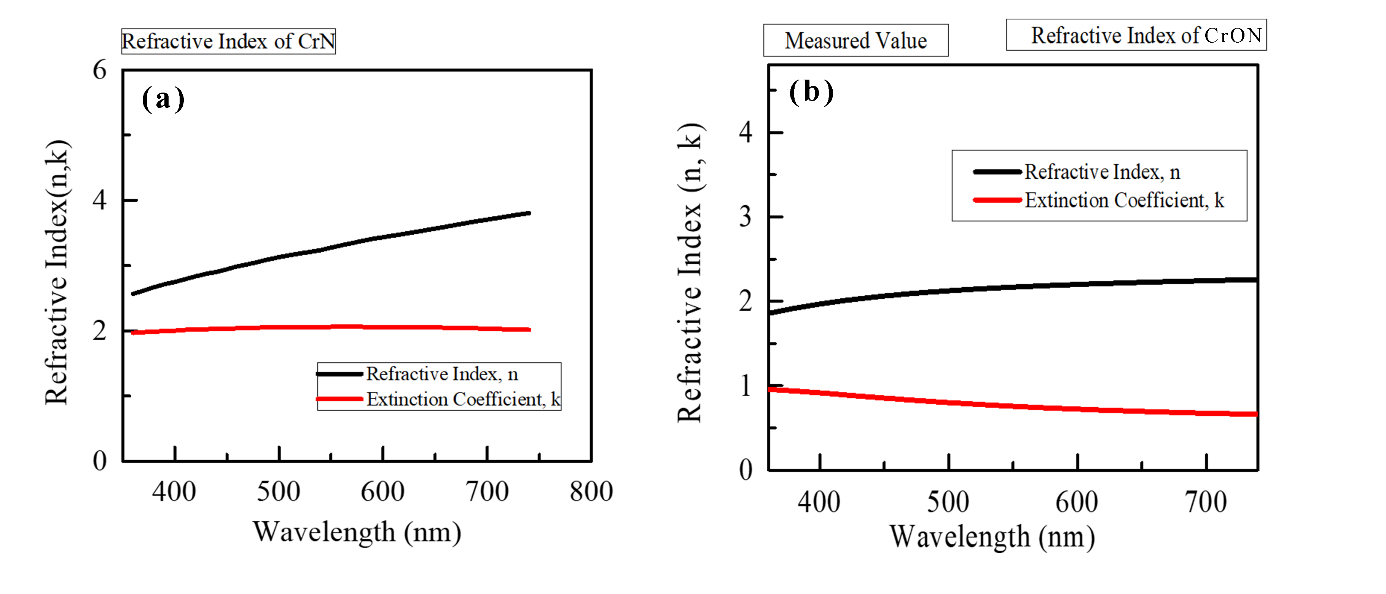
**

Fig. S1. The complex refractive indices of the CrN and CrON.

**
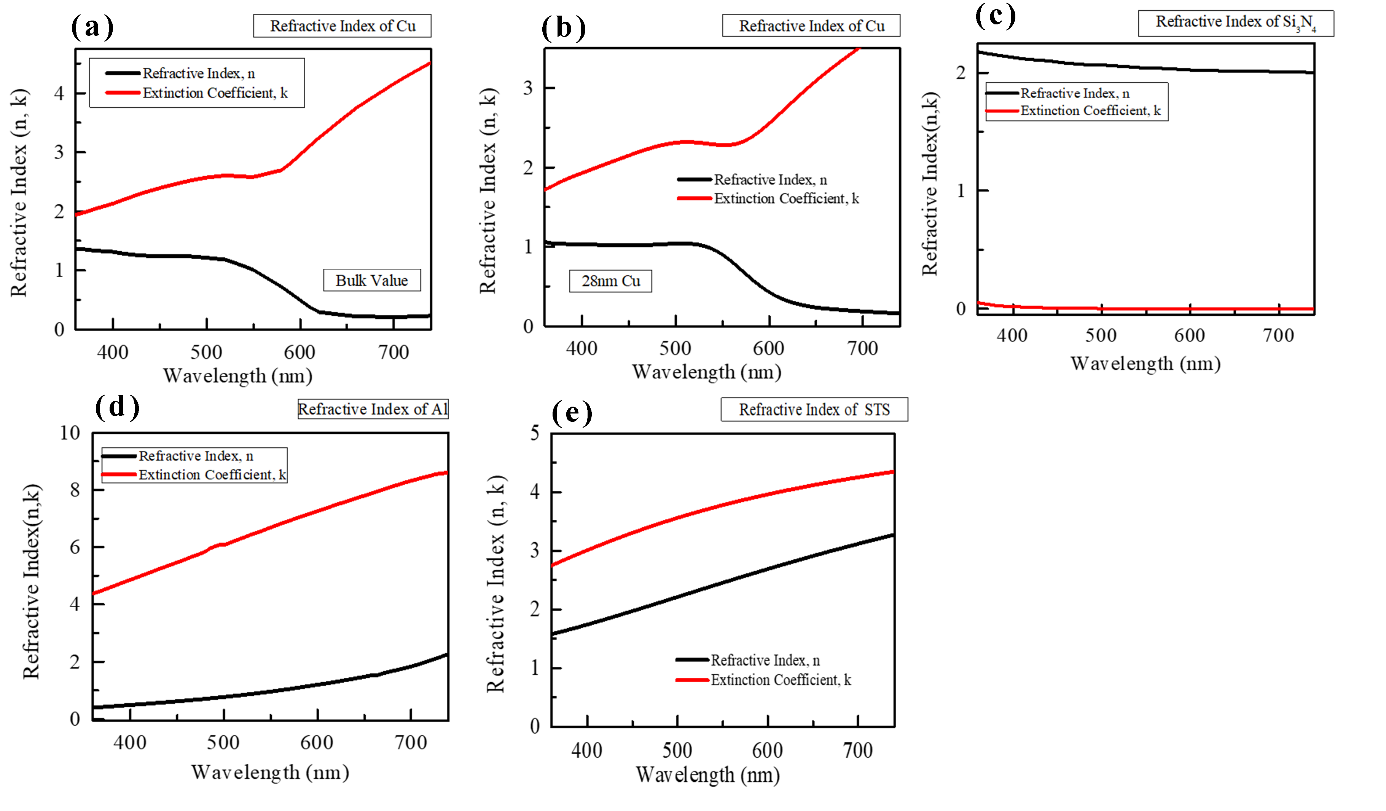
**

Fig. S2. The complex refractive indices of (a) bulk Cu, (b) 28 nm Cu thin film, (c) Si_3_N_4_ (d) Al and (e) stainless steel.

**
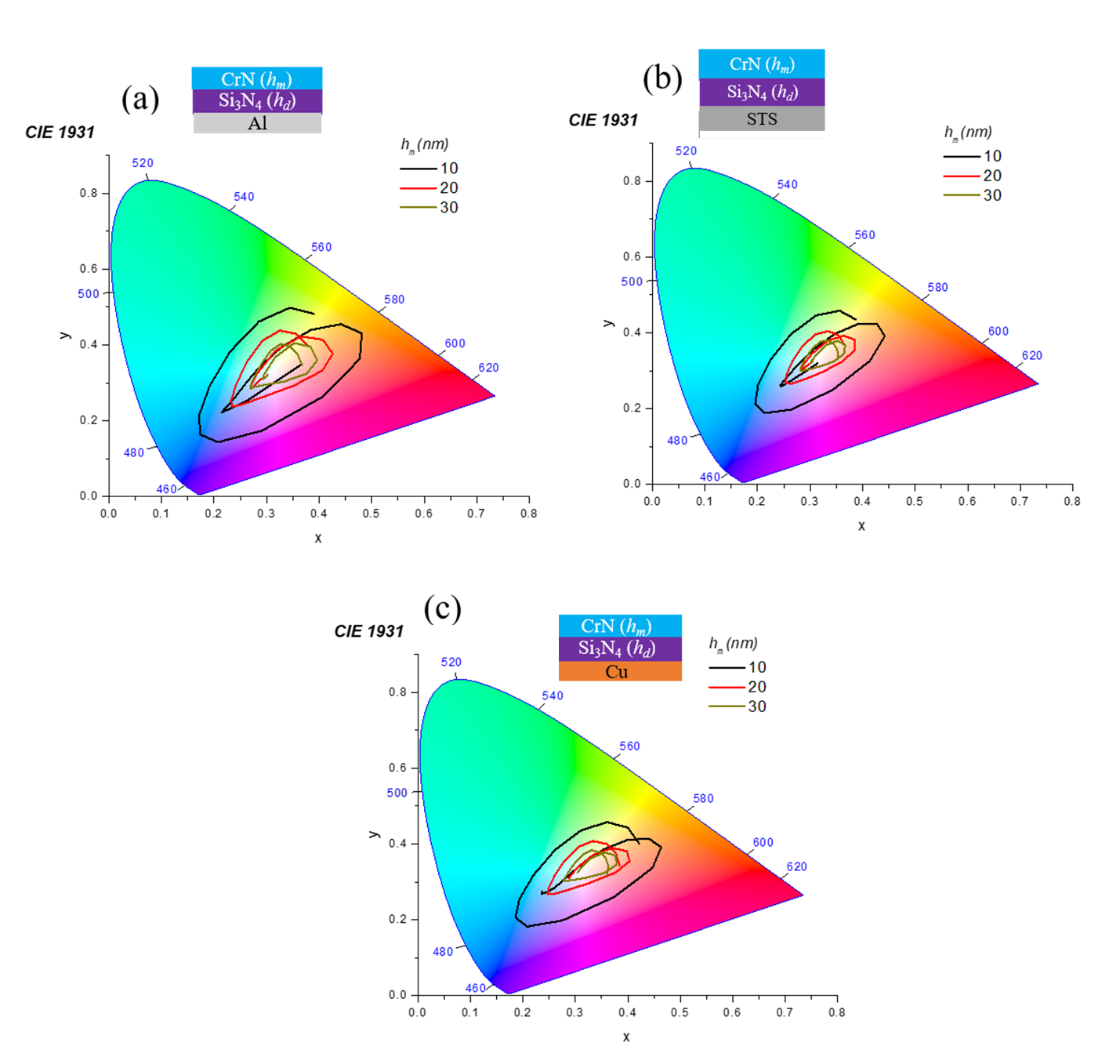
**

Fig. S3. CIE 1931 chromaticity diagram illustrating the CIE coordinates of the simulated (a) CrN/Si_3_N_4_/Al, (b) CrN/Si_3_N_4_/STS and (c) CrN/Si_3_N_4_/Cu structures as a function of ℎ_𝑚_ and *h_d_*; ℎ𝑚; *hm*=10–30 nm and ℎ_𝑑_=30–250 nm.


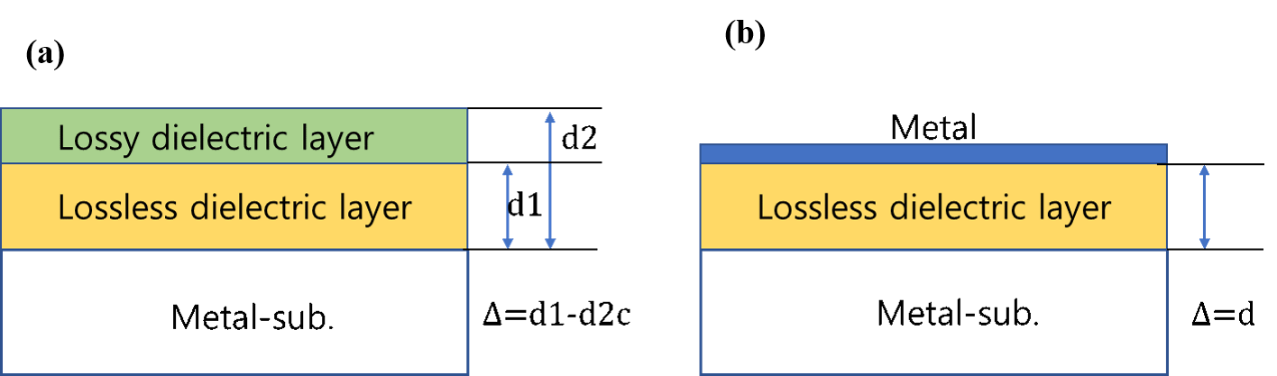


Fig. S4. Schematics to explain the absorption of lossy dielectric based structures compared with typical MIM structures.


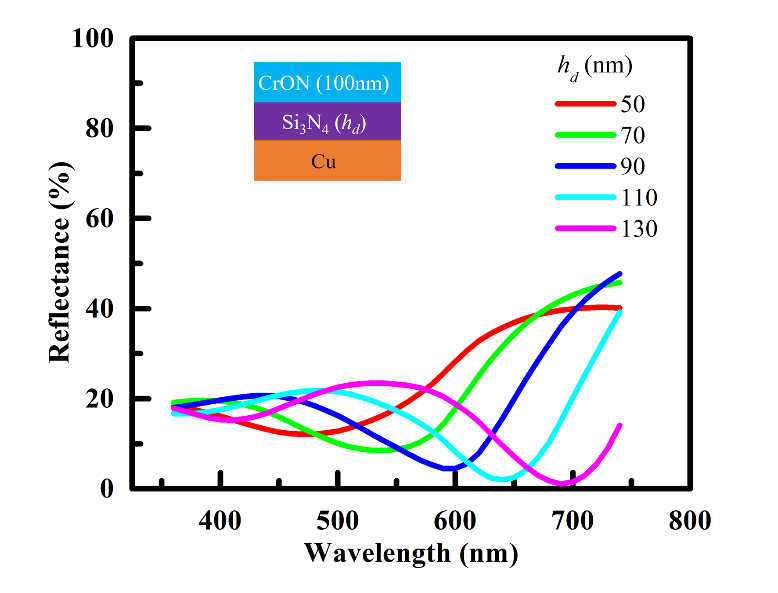


Fig. S5. The calculated reflectance spectra depending on the wavelength for the CrN(100nm)-Si_3_N_4_(*h_d_*)-Cu(100nm) structures.


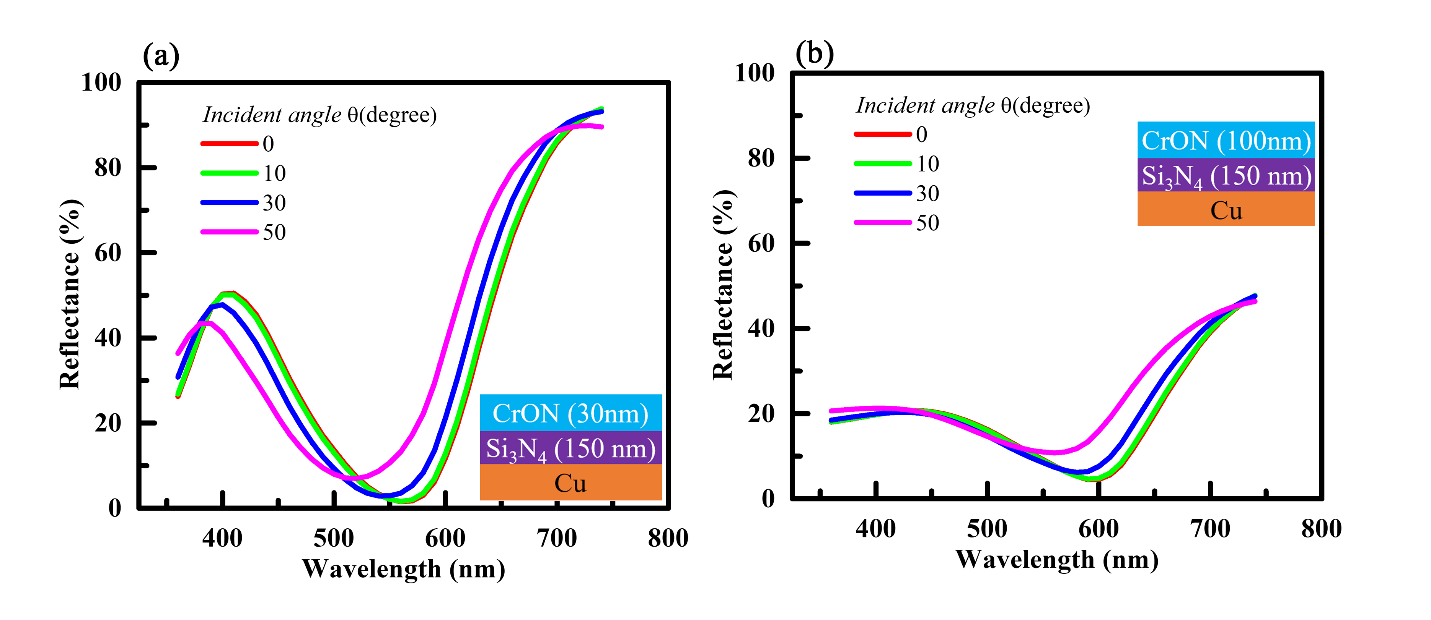


Fig. S6. The effect of incident angle on the reflectance spectra varying wavelength for (a) CrON(30nm)/Si_3_N_4_(150nm)/Cu and (b) CrON/Si_3_N_4_(150nm)/Cu structures.


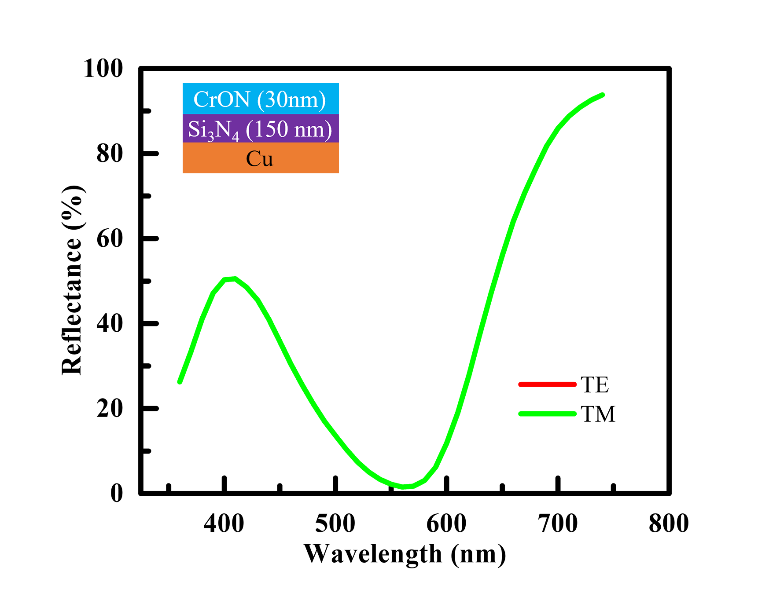


Fig. S7. The dependence of the calculated reflectance spectra varying wavelength with transverse electric (TE) and transverse magnetic (TM) polarization directions of incident light for the CrON(30nm)/Si_3_N_4_(150nm)/Cu structures. The calculated reflectance spectra of the CrON based structures are the same depending on the TE or TM direction of the incident light.

Table S1. Data for the absorption-wavelength and R_min_(%) for various *h_d_* in CrN(100nm)-Si_3_N_4_(*h_d_*)-Cu(100nm) structures.

| *h_d_*(nm) | λ(abs.) | R_min_(%) |
| --- | --- | --- |
| 50 | 470 | 12.0 |
| 70 | 540 | 8.50 |
| 90 | 595 | 4.41 |
| 110 | 640 | 2.35 |
| 130 | 690 | 1.30 |

Table S2. Data for the absorption-wavelength and R_min_(%) for various incident angles in CrN(30nm)-Si_3_N_4_(150nm)-Cu structures.

| *Incident angle, θ (degree)* | λ(abs.) | R_min_(%) |
| --- | --- | --- |
| 0 | 560 | 1.50 |
| 10 | 560 | 1.50 |
| 30 | 545 | 2.97 |
| 50 | 515 | 6.95 |

Table S3. Data for the absorption-wavelength and R_min_(%) for various incident angles in CrON(100 nm)/Si_3_N_4_(90nm)/Cu structures.

| *Incident angle, θ (degree)* | λ(abs.) | R_min_(%) |
| --- | --- | --- |
| 0 | 595 | 4.50 |
| 10 | 595 | 4.74 |
| 30 | 584 | 6.35 |
| 50 | 557 | 10.95 |
